# Effectiveness of a primary care based complex intervention to promote self-management in patients presenting psychiatric symptoms: study protocol of a cluster-randomized controlled trial

**DOI:** 10.1186/1471-244X-14-2

**Published:** 2014-01-03

**Authors:** Thomas Zimmermann, Egina Puschmann, Martin Ebersbach, Anne Daubmann, Susanne Steinmann, Martin Scherer

**Affiliations:** 1 Department of Primary Medical Care, Center for Psychosocial Medicine, University Medical Center Hamburg-Eppendorf, Martinistr. 52, Hamburg 20246, Germany; 2 Department of Medical Biometry and Epidemiology, Center for Experimental Medicine, University Medical Center Hamburg-Eppendorf, Martinistr. 52, Hamburg 20246, Germany; 3 Institute for General Practice, Hannover Medical School, Carl-Neuberg-Str. 1, Hannover 30623, Germany

**Keywords:** General practitioner, Advanced practice nurse, Primary care, Self-Management, Anxiety, Depression, Somatization, Somatic symptom disorder, Cluster-randomized clinical trial, Health services research, Supplementary nursing care

## Abstract

**Background:**

Anxiety, Depression and Somatoform (ADSom) disorders are highly prevalent in primary care. Managing these disorders is time-consuming and requires strong commitment on behalf of the General Practitioners (GPs). Furthermore, the management of these patients is restricted by the high patient turnover rates in primary care practices, especially in the German health care system.

In order to address this problem, we implement a complex, low-threshold intervention by an Advanced Practice Nurse (APN) using a mixture of case management and counseling techniques to promote self-management in these patients. Here we present the protocol of the “**S**elf-**M**anagement Support for **A**nxiety, **D**epression and **S**omatoform Disorders in Primary Care” (**SMADS**)-Study.

**Methods/Design:**

The study is designed as a cluster-randomized controlled trial, comparing an intervention and a control group of 10 primary care practices in each case. We will compare the effectiveness of the intervention applied by an APN with usual GP-care. A total of 340 participants will be enrolled in the study, 170 in either arm. We use the Patient Health Questionnaire-German version (PHQ-D) as a screening tool for psychiatric symptoms, including patients with a score above 5 on any of the three symptom scales. The primary outcome is self-efficacy, measured by the General Self-Efficacy Scale (GSE), here used as a proxy for self-management. As secondary outcomes we include the PHQ-D symptom load and questionnaires regarding coping with illness and health related quality of life. Outcome assessments will be applied 8 weeks and 12 months after the baseline assessment.

**Discussion:**

The SMADS-study evaluates a complex, low threshold intervention for ambulatory patients presenting ADSom-symptoms, empowering them to better manage their condition, as well as improving their motivation to engage in self-help and health-seeking behaviour. The benefit of the intervention will be substantiated, when patients can enhance their expected self-efficacy, reduce their symptom load and engage in more self-help activities to deal with their everyday lives. After successfully evaluating this psychosocial intervention, a new health care model for the management of symptoms of anxiety, depression and somatoform disorders for ambulatory patients could emerge, supplementing the work of the GP.

**Trial registration:**

Clinicaltrials.gov Identifier: NCT01726387

## Background

Anxiety disorders, depression and somatoform disorders (hereafter referred to as ADSom disorders) belong to the most common mental disorders in primary care [1]. ADSom disorders contribute to a substantial utilization of the health care system [2]. Also, ADSom disorders cause significant direct and indirect health care costs [3].

### Mental disorders – an urgent problem in primary health care

There is no controversy about the pivotal role of primary care in treating these mental conditions: most patients with ADSom symptoms are primarily and often exclusively seen by their general practitioner (GP) [4-6]. A survey conducted by the Bertelsmann Foundation [7] revealed only 13% of patients with psychological complaints over the past 12 months were seeking solely specialized care. Moreover, ADSom-disorders are frequently associated with, or sometimes masked by, somatic disorders, therefore, an additional somatic medical examination by the GP is necessary [8]. Not least, the World Health Organization recommends the integration of mental health care into primary care. It “enhances access, promotes respect of human rights, is affordable and cost effective, and generates good health outcomes” [9].

However, targeting the many problems these patients convey exceeds the resources of most GPs. Also, high patient turnover rates in primary care inevitably limit contact time between GP and patients: In Germany, an average of 18 primary-care-contacts per year per patient in 2007 [10], even 35–54 contacts per year for multi-morbid patients [11], render it impossible to discover and unveil backgrounds and/or motifs of the psychological symptoms and complaints. Regular consultations focus mainly on acute care, thus falling too short to enhance and support self-management skills of the patients. Professional self-management support is beyond the scope of usual GP-care, although self-management support through patient education and counseling is a cornerstone of mental health care [12].

The German social security system offers quite a few different services for patients in psychosocial need: helpdesks, helplines, publicly-funded self-help groups, counseling services, community based social-psychiatric support, rehabilitation services, re-integration services after long sickness leaves, and if nothing else: psychotherapy. Even though the services are there, more often than not ADSom-patients are not able to help themselves by taking the next step - accessing a service. There is paperwork to do, contacting, networking, information seeking and so on – tasks patients with psychological complaints have a hard time executing. Symptoms prevent the utilization of available services [13]. Finally, there is a lack of coordination amongst these services as they are offered in an unsystematic and erratic way. Depending on the information they have, GPs recommend certain services to their patients, but quite a few are reluctant to get involved in the time-consuming, usually not reimbursed methods of accessing these services. In order to supplement patient management, we introduce a specially trained Advanced Practice Nurse (APN) working alongside a GP in the practice.

Several trials support this collaborative care model. Health care assistants helped to reduce patients’ depressive symptoms in the PROMPT-Study [14]. Elderly patients showed long-term improvement in their depressive symptomatology in the IMPACT-trial [15]. A program to manage depression interprofessionally proved to be effective in the INDI-trial [16].

A recent Cochrane review concludes: “Collaborative care is associated with significant improvement in depression and anxiety outcomes compared with usual care, and represents a useful addition to clinical pathways for adult patients with depression and anxiety” [17].

In recent years, Germany has begun to gradually change its legislation to allow health care professionals like APNs to deliver services for patients. The law now facilitates the delegation of a wide variety of health services from a GP to a health care assistant or an APN. In spring 2012 a directive for the delegation of medicinal work to other health care professions (“Heilkundeübertragungsrichtlinie”) was put into action by the Joint Federal Commission, a ruling body for health care services in Germany [18].

Based on that directive we implement a complex, low-threshold intervention by an APN using case management and counseling techniques to support ADSom-patients. The APNs will help the patients to better understand their symptoms and complaints, identify stressors and resources, as well as create goals to be attained in the course of the intervention.

Here we report the study protocol of the “**S**elf-**M**anagement Support for **A**nxiety, **D**epression and **S**omatoform Disorders in Primary Care” (**SMADS**)-Study.

### Objective

The objective of the study is to examine the effects of counseling and case management on the concept of self-efficacy. We want to investigate whether an APN, collaborating with a GP, addressing the psychosocial needs of ADSom-patients can enhance the patients self-efficacy (a proxy for self-management) compared to usual care.

Furthermore, we want to know if an APN decreases patients’ symptom loads and psychosocial burdens, and increases their quality of life, while reducing health care utilization. A further assignment of the APN is to implement case management elements into the primary care practice. After all, we want to estimate whether cooperation between an APN and a GP does improve health care services for these patients.

The study is part of “Psychenet - Hamburg Network for Mental Health” (http://www.psychenet.de/en.html) - a network in the Hamburg region consisting of more than 60 scientific and medical institutions, counseling centers, governmental authorities, private companies, health insurances as well as patients’ and relatives’ associations. Its purpose is to work on testing innovative care models which aim to make decisive improvements to the prevention, diagnosis and treatment of people with mental illnesses in the region [19].

## Methods

### Study design

We set up an open label, cluster-randomized controlled trial. We randomly allocate participating general practices to either the interventional arm, in which a nurse-led collaborative care model is implemented, or to the control arm in which the GP continues his/her usual routine care treating ADSom-patients.

**Figure 1:**
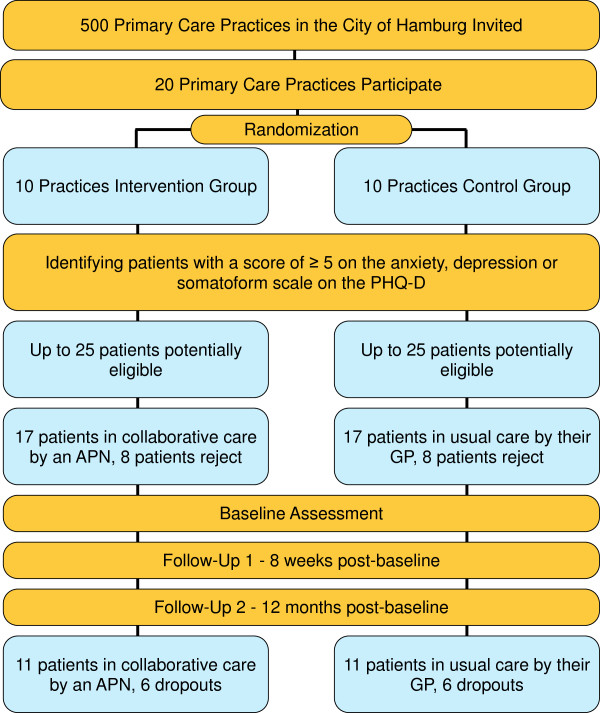
CONSORT flowchart for recruitment of practices and patients (projected).

The APN will work directly in the primary care practices. All participants will be assessed at baseline, 8 weeks post-baseline and 12 months post-baseline. The scheduled time for providing services in the interventional primary care practices is 12 months. In the control practices, the patients obtain the „usual care“ by the GP. In this arm, the course of the disease and the utilization of GPs or other professions will be documented. The interventions will take place in the general practices, the APNs having an own office at their disposal. The practice and the APN arrange a fixed weekday on which the nurses work in this particular practice. Figure 1 illustrates patient flow of the SMADS-study.

### Ethics approval

Ethics approval was obtained from the Ethics Committee of the Hamburg Medical Association in October 2012, approval number PV4106.

### Recruitment of the primary care practices

The city of Hamburg is a large metropolitan area with a population of 1,8 million. There are about 1000 primary care practices, about 70% of which are owned and operated by a single GP and his/her practice assistants, while about 30% are group practices.

**Figure 2:**
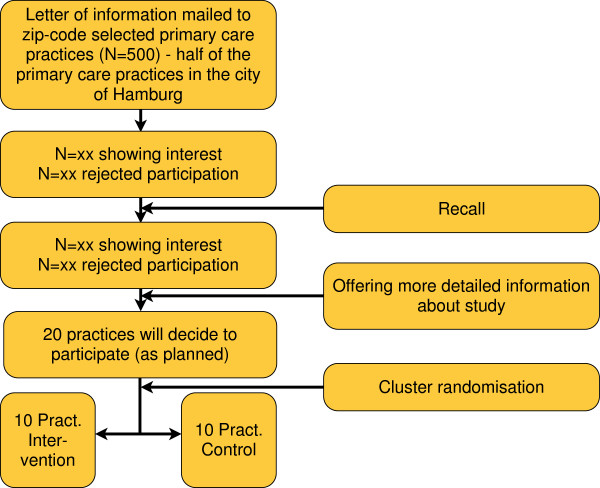
Planned recruitment of primary care practices.

The recruitment of the 20 GP-practices (10 in the interventional arm, 10 in the control arm of the study) will occur through a letter of invitation mailed out to 500 primary care practices in five districts of Hamburg city, according to the zip code. If they show interest, the trial will be introduced in more detail. Practices which don’t respond at all will be selectively recalled to ask for the reasons (see Figure 2).

### Randomization

Participating practices will be randomly allocated to either arm of the trial. A biometrician (AD), not involved in the field work, randomly selects the practices for the treatment- and the control-arms of the study.

### Modules of the complex, psychosocial intervention

Promoting self-management is the target of the planned complex intervention. Strategies of self-management support are based on knowledge and information transfer to increase health literacy and facilitate the development of skills, as well as promoting the use of available health resources [12]. Self-management support promotes control and responsibility, thus strengthening the patients’ confidence in their own ability to manage his or her psychological symptoms, as well as their impact on his or her daily life [20].

More explicitly, the aim of any self-management in psychological complaints is to empower the patient:

• to actively pursue the challenges in dealing with mental symptoms and syndromes,

• to develop an adequate strategy for dealing with the negative side effects,

• to set up an appropriate symptom management,

• to use problem solving skills in order to manage daily life, and

• to support the patients and their families on their way to become qualified (lay)-experts in their own right, as well as

• to prevent chronification, thus to contribute to the prevention of personal dependency and home care [21].

**Figure 3:**
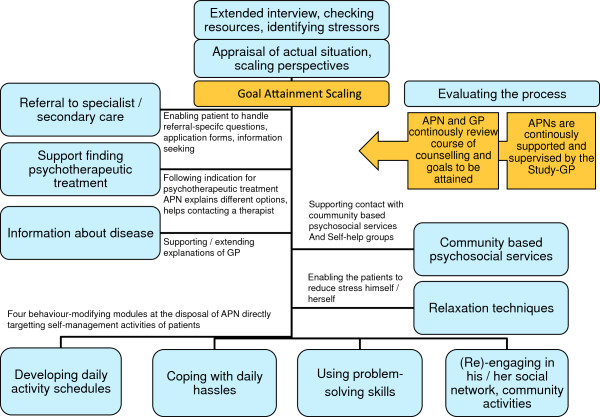
Modules of the complex intervention.

The intervention is comprised of several modules to engage the patient in better self-management and self-care (Figure 3):

1) Support in finding psychotherapeutic treatment: Following the indications for psychotherapeutic treatment, having the APN explain different options, support in contacting a therapist,

2) Information about disease: Supporting / extending explanations of the GP, plus four behaviour-modifying modules at the disposal of the APN which directly target the self-management activities of patients such as:

3) Developing daily activities schedules,

4) Coping with daily hassles,

5) Using problem-solving skills,

6) (Re)-engaging in his / her social network, and community activities, as well as

7) Learning relaxation techniques to enable the patients to reduce their stress levels on their own and

8) Supporting the patient in making contact with community based psychosocial services and self-help groups.

Every single intervention plan will be customized individually for each patient according to the specific needs and the resources a patient offers. Therefore, an APN’s prior task is to explore patients’ resources for self-management, symptom control and self-care. An APN has to gain information which situations and conflicts may affect the patients’ exposure to stress the most.

Stimulating resource-oriented behaviour modification will be assessed using Goal Attainment Scaling [22] – a measure for a single goal to be attained by the patient, negotiated and decided upon by both the APN and the patient. In cooperation with the patient, the APN explores the motivation for change, gathering goals for the patients using the **SMART**-criteria [23]: **S**pecific, **M**easurable, **A**chievable, **R**ealistic/**R**elevant, **T**imed. At the end, the measure allows a comparison between a reference and an actual value.

Finally, there is the collaborative part of the intervention as the APN’s work is undertaken in close coordination with the GP:

• The GP documents which module he or she recommends for the intervention plan.

• Appointments are made to share information between GPs and APNs over the course of the intervention, discussing the progress, and creating ways to rescale the intervention plan.

• Evaluation: Throughout the intervention, the process will be evaluated by everyone in the triad: the patient, the GP, and the APN.

• In the 12-month-follow-up patients, as well as the GPs, will be interviewed to collect information about the sustainability of the intervention.

Backing up the collaborative care team are two study scientists: EP, the study GP and psychotherapist, is preparing the interventions as well as continuously supporting and supervising the APNs. The study psychologist (TZ) is organizing the set-up in the primary care environment, connecting APNs, GPs and practice staff, as well as helping to debrief the nurses at times.

### Advanced practice nurse

We decided to employ nurses experienced in the management of patients with psychiatric symptomatology or psychosocial needs, preferably having either a background in primary care or a bachelor of science in nursing. Closely monitored by the study-GP, the APNs go through a training program incorporating written documentation and practical training, live enactments with simulated patients, and sitting in on therapy in psychiatric wards or a psychosomatic outpatient clinic.

Since we have created the job profile with part-time potential, an APN will need to be quite mobile and flexible. They are supposed to rotate between up to five different practices, handling a different working environment and different working hours every weekday.

### Study population and recruitment

The study is conducted in the city of Hamburg. Citywide, half of the GPs are asked to participate. Additionally, we will recruit GPs by introducing the project in quality circles and contacting the Hamburg Association of General Practitioners. As we build up the APN staff step by step we include practices in a similar mode. The process of recruiting practices is ongoing.

Inclusion criteria for practices are:

• Willingness to participate in the study regardless of randomization to the intervention or control arm,

• One single private room at scheduled times for the APN to deliver the intervention in a protected environment,

• No psychotherapeutic treatment within the practice, neither by the GP him/herself nor by any other professional in the practice,

• No participation in other studies of “Psychenet: Hamburg Network for Mental Health”.

The patient recruitment is based on an assessment taking place in the practice on a particular screening day. As the capacity of the APN is limited, and we don’t want to create waiting lists, it is required to recruit patients into the study adjusted to the APN’s treating capacity.

The first part of the assessment is done by the GP: All patients who are seen by the doctor on the screening day, will be put on a chart and checked for inclusion criteria.

Eligibility criteria for patients are:

1) PHQ ≥ 5 on the anxiety, depression or somatoform scale,

2) Age: 18–65 years old,

3) Literacy (German),

4) Fully able to give consent,

5) Sufficient auditory and visual capabilities,

6) Currently no psychotherapeutic treatment.

### PHQ-D-assessment

Patients found eligible will be informed about the study. The patient will receive written and verbal information from the GP. The information covers the aims and procedures of the study, the selection of participants, the data collection, processing and storage, as well as possibilities for cancellation. In case these terms are accepted, the participants will have to sign an informed consent form to participate in the study. Besides that, patients have to agree to release their physician of his/her medical confidentiality allowing the APN and the GP to exchange information. Patients will be asked to complete the “Patient Health Questionnaire-D (PHQ-D)” [24] and other instruments introduced later in this paper.

The target population for the intervention are adults aged 18 to 65 scoring ≥5 on any of the three symptom scales that are incorporated in the PHQ-D: Anxiety, using the seven items of the General Anxiety Disorder-Scale (GAD-7); Depression, using the nine items of the PHQ-9 depression scale; or somatoform symptoms which are checked by the fifteen-item somatization scale of the PHQ, PHQ-15.

Psychiatric symptoms fluctuate on a daily basis and overlap between the disorders [8]. Thus, we expect many patients to score above the cut-off on any of the scales. A score as low as ≥5 to gain access to the counseling service in the GP practice emphasizes the low-threshold approach of the intervention. When considering prevention, early support may impede a chronic condition later on.

### Exclusion criteria

We limit the exclusion to the negation of the inclusion criteria. Also, participation in other studies of our department will exclude a patient from this study. Moreover, we didn’t enroll terminally ill or immobile patients, or patients who are not regulars in the practice.

### Sample size/power calculation

**Figure 4:**
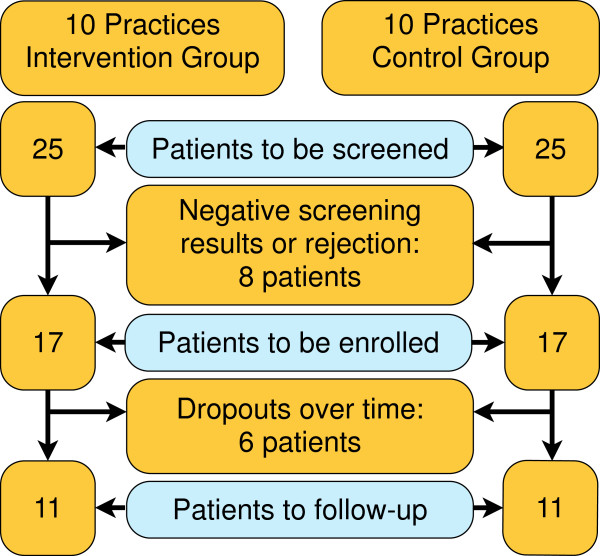
Recruitment and flow of patients (projected).

We calculated a sample size of 220 patients based on an expected difference between the intervention and the control group of 2.7 points and a common standard deviation of 5.4 points on the General Self-efficacy scale as the primary outcome 12 months post baseline. assuming an intra-cluster correlation of 0.05, we will attain a sufficient study power of 80%. For that we need a minimum of 20 randomized clusters, with an average cluster size of 11 patients. Assuming a drop-out rate of 33% on the patient level, we have to recruit 340 patients (on average 17 patients per cluster). The planned numbers and the patient flow are shown in Figure 4.

### Primary outcome measure

The primary outcome measure of the SMADS-study is self-efficacy. This measure will be assessed by the General Self-Efficacy Scale (GSE-Scale), [25].

Perceived self efficacy is an important prerequisite for successful self-management [26]. The construct, theoretically and empirically [20] well founded, was originally developed by Albert Bandura in the 1970s. Bandura defines self-efficacy as “people’s beliefs about their capabilities to produce designated levels of performance that exercise influence over events that affect their lives” [27]. Based on improved self-efficacy patients can regain control of their own lives, gaining new confidence in their ability to perform a task. Yet, as Battersby et al. wrote, “self-efficacy is not a process; it is an intermediate outcome or mediator of a patient’s adoption of self-management behaviours and health behaviour changes” [28].

The GSE-questionnaire operationalizes the psychological construct „self efficacy“, consisting of 10 items ranging from 1 = Not at all true, 2 = Hardly true, 3 = Moderately true to 4 = Exactly true. The GSE is a valid, theoretically driven, globally used measurement for the assessment of self-efficacy and has been translated into 31 languages. The scale measures a general sense of perceived self-efficacy. It predicts the ability to cope with everyday life, as well as the ability to adapt after experiencing all kinds of stressful life events [29].

### Study hypotheses

We hypothesize, the APN will improve the self-efficacy expectations of the patients in the intervention group by about 2.7 points assuming a common standard deviation of 5.4 points. A significant increase in this measure indicates a better ability to perform the broad range of self-management techniques. A patient who is more convinced of his or her own chance to have impact on one’s own life is better prepared to build up resilience against stressors in life.

### Secondary outcome measures

As secondary outcome measures we assess the symptom load using the PHQ-D. The PHQ-D is a well established instrument to screen patients for anxious, depressive and somatoform symptoms. It has good validity and prognostic capabilities [24]. We decided against the use of the PHQ-D as the primary outcome, even though we use it as a screening tool to include patients. This is justified by the supreme goal of the study: Supporting self-management, measured indirectly, using self-efficacy as a proxy.

In addition, patients will be assessed on their health related quality of life, answering the EQ-5D questionnaire. The EQ-5D, a 5-item-questionnaire, developed by the EuroQoL-group (http://www.euroqol.org), is comprised of five dimensions: mobility, self-care, usual activities, pain/discomfort and anxiety/depression. A visual analogue scale evaluates the patients self-rated health on a vertical scale where the endpoints are labeled ‘100 - Best imaginable health state’ and ‘0 - Worst imaginable health state’. The EQ-5D has satisfying psychometric properties. There are published reference values for the general population [30]. Furthermore, coping will be assessed with the “Coping with Illness scale” [31]. It assesses a broad range of cognitive, behavioural and emotional aspects of coping with an illness. Investigators use the short version.

Further evaluations include the analyses of cost-effectiveness, the subsequent number of sickness leaves, and the utilization of the health care system in the year post-baseline.

### Statistical analyses

For the primary outcome we evaluate the changes in comparison to the baseline GSE after 12 months. A linear mixed model will be calculated for the difference between the intervention and control groups. The variable “group” will be considered a fixed effect while the practice will be considered a random effect under the control of the baseline covariates (baseline values of GSE and other confounders like PHQ-D, age, gender, education, utilization of the health care system on patient level, as well as age, gender, status of practice (single or group) on practice level. The two-sided α-level was set to 0.05.

The analyses of the primary outcome will be based on an intention-to-treat-(ITT)-analysis: All patients enrolled into the study will be analyzed at follow-up. All drop-outs not having withdrawn their consent will be asked again to take part in the final assessment 12-months-post-baseline. If patients reject this invitation or cannot be reached at all, their values will be imputed using the last-observation carried forward (LOCF) method.

The robustness of the results will be investigated performing sensitivity analyses with different methods of imputing missing values.

The same procedures will be applied for the PHQ-D, the EQ-5D and the „Coping with Illness scale“ as secondary outcomes. There will also be analyses of the observed cases (OC). These analyses will include only those patients, who did not drop out and completed the final assessment.

### Handling of dropouts

We are accounting for 2 different groups of dropouts:

• Patients declaring to abandon the intervention or repeatedly missing appointments, and

• Patients cancelling their consent, thus, withdraw from the study.

All dropouts, who will not withdraw their consent will be contacted again 12 months post-baseline to get a chance to answer the study questionnaire. Dropouts are accounted for in the intention-to-treat-analyses.

### Methods against bias

Selection bias will be minimized by the randomization of the practices. The standard recruiting procedure (establishing a screening day in the practice) also tries to keep the selection bias checked. In order to collect the required data for a CONSORT flow chart (for clustered trials), the full recruitment process will be documented.

### Public registration

Before starting, the trial was publically registered with an internet based trial archive, clinicaltrials.gov (NCT01726387). http://clinicaltrials.gov/show/NCT01726387.

### Data monitoring

An independent monitor (Institute for General Practice, Hannover Medical School) will conduct data monitoring to ensure high quality data in adherence to the study protocol. All the paper-pencil-documentation the APNs produce (memos, process evaluation etc.) will be stored in the GP-practice and will be made available for the practice documentation.

### Detection bias

Practices in the control arm will have the chance to employ an APN after finishing the randomized controlled trial in order to avoid a possible lack of motivation for recruitment and documentation. The APNs will be working in these practices for another 12 months. Furthermore, control practices will get the same financial compensation for their engagement as the GPs in the intervention arm.

### Stopping rules

As the planned intervention does not introduce any specific therapeutic changes but focuses on resources and self-management, negative effects are very unlikely. Anyhow, patients presenting psychological complaints can deteriorate – so there is a standardized module implemented to check for suicide risk in patients. The collaborative care setting in the GP-practice protects the patient and the APN in case of an immediate aggravation. The following stopping rule will be put into action: If patients’ behaviour threatens the APNs or themselves, the APN will refer them back to the GP or reject the intervention in the first place.

### Quality assurance and safety

IT, data management and quality assurance will be provided by the Institute for General Practice, Hannover Medical School. Quality assurance consists of procedures for the prevention of insufficient data quality, the detection of inaccurate or incomplete data and actions to improve data quality, e.g. user training sessions, automatic plausibility and integrity checks within the remote data entry system and data error reports. In addition, the study center will regularly receive feedback in the form of quality reports. In addition a random sample of paper questionnaires (5%) will be compared with the data entries in the database. Adverse events will be monitored and reported.

## Discussion

This cluster-randomized controlled trial implements a low threshold, complex, psychosocial intervention for primary care patients reporting at least moderate symptoms of anxiety, depression or somatization. It contains several innovative features:

• It brings an APN into a GP-practice creating a confidential atmosphere as the patient and his or her problems, symptoms, and complaints are already known within the practice.

• It attempts to establish a new professional profile, the APN, into the German health care system.

• It creates a collaborative care model widely acknowledged to be beneficial for patients with psychosocial needs.

• It gains additional knowledge about the needs of patients who fail to climb some of the high barriers that lie between them and the access to a psychosocial service.

• It puts several of the evidence-based principles for implementing self-management support in primary care into action that were assembled by Battersby et al. [28] in their review of reviews covering 83 meta-analyses. These include brief targeted assessments, evidence-based information to guide shared decision-making, the use of a nonjudgmental approach, collaborative priority and goal setting, and collaborative problem solving, just to name a few.

Also, the trial will offer answers for these questions:

• Is this complex intervention effective in enhancing self-efficacy, thus empowering the patients to a better self-management?

• Which beneficial or obstructive conditions go along with the establishment of a new health professional?

• Eventually, we will be taught by the patients whether this supplementary health care service supports them in feeling better off: Is it what they really need?

The study investigates a new type of collaboration between GPs and APNs for patients with symptoms of anxiety and depression as well as for patients with somatoform symptoms in ambulatory care. The objective of the intervention is to empower the patients to enhance self-efficacy and activate a resource-oriented self-healing process through better self-management and self-care. We want to examine whether or not and to what extent collaborative care between a family doctor and a nurse can mitigate the psychological complaints of the patients.

## Competing interests

The authors declare that they have no competing interest.

## Authors’ contributions

TZ, EP, MS designed the trial and planned the protocol. AD contributed the sample size calculation and the randomization, EP planned the modularized complex intervention and ME contributed to several intervention modules. SS set up the remote data entry system and the concept for data quality assurance. TZ planned and drafted this paper. MS substantially revised it. All authors endorsed the publication. All authors read and approved the final manuscript.

## Pre-publication history

The pre-publication history for this paper can be accessed here:

http://www.biomedcentral.com/1471-244X/14/2/prepub
